# Atypical Hyaluronic Acid-Induced Vascular Occlusion Masked by a Hematoma: Ultrasound-Guided Diagnosis and Management

**DOI:** 10.7759/cureus.108472

**Published:** 2026-05-08

**Authors:** Seline Rasheed, Iman Itani

**Affiliations:** 1 Dermatology, Syrian Private University, Damascus, SYR; 2 Dermatology and Aesthetic Medicine, Lueur Aesthetic Clinic, Dubai, ARE

**Keywords:** filler complications, hematoma, hyaluronic acid filler, hyaluronidase, nasolabial fold, ultrasound-guided, vascular occlusion

## Abstract

Hyaluronic acid (HA) fillers are widely used in aesthetic practice as they are generally considered safe and effective. However, vascular occlusions remain a rare but serious complication that can cause tissue ischemia and necrosis if they are missed. Recently, with the challenges of early diagnosis, ultrasound has emerged as a valuable tool in identifying vascular compromise through Doppler flow assessment and visualization of filler deposits, supporting both the diagnosis and management of these events. We present a case of vascular occlusion following HA filler injection to the nasolabial fold and the upper lip, presenting with swelling, blanching, and an atypical, exaggerated hematoma. Initial management at the referring clinic including high-dose hyaluronidase and lidocaine administration was insufficient, followed by the development of a hematoma, which raised the concern for an incomplete resolution of symptoms and complicated accurate assessment of progression and severity. Further evaluation using ultrasound precisely confirmed vascular occlusion of the left angular and left superior labial arteries and guided targeted hyaluronidase administration, allowing for immediate restoration of vascular flow and progressive clinical improvement, resulting in a highly effective approach compared to blind, high-dose treatments. This case highlights the importance of accurately diagnosing vascular occlusions, even when the presentation is not classic. Early recognition and careful monitoring after filler injections are essential to prevent progression and reduce complications. Effective management can significantly improve outcomes, and the use of ultrasound adds a great advantage by improving visualization of vascular structures and enabling more precise and targeted treatments.

## Introduction

In recent years, with the rise of certain aesthetic looks, the demand for hyaluronic acid (HA) dermal fillers has increased greatly, due to their minimally invasive nature, effectiveness, and safety in comparison to other surgical alternatives [1,2]. Dermal fillers are mainly used in facial rejuvenation to enhance facial harmony, restore lost volume, or even augment certain facial features [3]. However, along with their widespread use, complications increase consequently; among which, vascular occlusions remain one of the most serious and threatening outcomes, whether due to intravascular injection, vascular compression, or embolic spread, with reported incidences of skin necrosis around 0.001% and vision-related complications with a rate of 1 in 5000 injections [1,4]. Although rare, vascular occlusions may lead to skin ischemia, tissue necrosis, and, in severe cases, irreversible vision loss [4].

Early recognition and management of vascular occlusions are critical and essential to prevent permanent damage to surrounding tissues. Current guidelines consider hyaluronidase as the cornerstone of treatment, with emphasis on timely and targeted administration to restore reperfusion [5]. However, the diagnosis of vascular occlusions is often based on clinical assessment, which may be challenging in certain scenarios, particularly when classical signs such as pain, blanching, livedo, delayed capillary refill, and skin discoloration are concealed or altered, thus delaying intervention [5,6].

Currently, with the increased complexity and the high potential for serious complications, ultrasound has become a valuable addition to aesthetic medicine, allowing for real-time and precise visualization of vascular structures, soft tissues, and filler deposits [6,7]. In the diagnosis and management of filler-induced complications, it can accurately identify compromised vessels and guide real-time intervention.

In this report, we present a HA filler-induced vascular occlusion in the perioral region. The initial management, which involved multiple hyaluronidase injections, resulted in hematoma formation, obscuring clinical evaluation and delaying diagnosis and treatment. The use of ultrasound allowed for accurate identification of the underlying vascular compromise and guided targeted treatment, leading to a complete resolution of symptoms without permanent damage. 

## Case presentation

A 35-year-old woman presented to our clinic with swelling, blanching, and skin discoloration after receiving a dermal filler injection to the nasolabial folds and the upper lip performed at an external clinic earlier the same day. Immediately after the procedure, the patient developed concerning symptoms suggesting that a vascular occlusion began to progress, and she was managed initially at the referring clinic before being transferred to our facility for further evaluation and treatment.

The patient had received a total of 0.6 ml of HA filler, and it was stated that during the injection of 0.1 ml of HA filler into the left nasolabial fold at the level of the piriform fossa using a 27G needle, immediate blanching was observed above the upper lip and at the injection site. However, this finding was initially ignored and considered a normal response, and the procedure was continued. Additional injections were administered to the right piriform fossa and superficial bilateral nasolabial folds using a 25G cannula, as well as the upper lip with a needle. Shortly after, blanching and pain extended across the left nasolabial fold, reaching the upper lip, nose, glabella, and the left periorbital region, corresponding to the distribution of the facial and angular arterial supply, along with delayed capillary refill.

Initial treatment at the referring clinic included administration of five cycles of hyaluronidase. The treatment was initially applied in a diffuse manner across the affected area using a flooding technique, followed by targeted application along suspected facial arterial pathways. A total of eight vials (3000 IU/vial x 8 = 24,000 IU), each diluted with 5 ml of normal saline, were administered, with massage applied after each cycle. In addition, the patient received Aspirin 400 mg, Zinnat 500 mg (oral cefuroxime), and a local injection of lidocaine for pain management. Partial improvement was noticed; however, the patient continued to experience delayed capillary refill and persistent blanching in the left buccal area, which led to the referral to our clinic.

**Figure 1:**
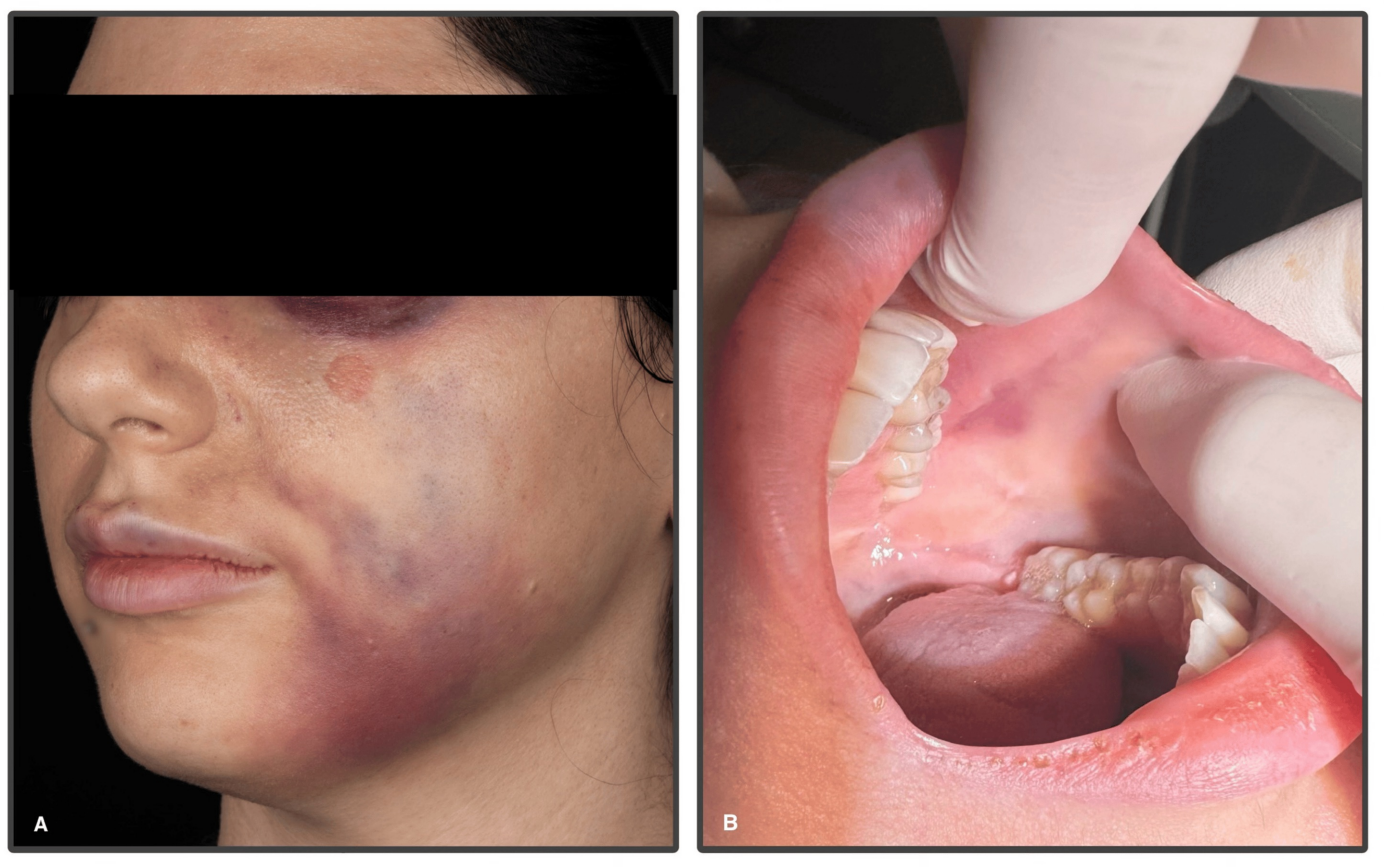
Initial clinical and intraoral presentation of vascular compromise. (A) Clinical presentation demonstrating blanching in the upper lip, left nasolabial and buccal area, swelling and severe bruising over the left periorbital region, left nasolabial fold region, upper lip, and lower face. (B) Intraoral examination demonstrating ecchymosis (bruising) of the inner buccal mucosa in the affected region.

The patient presented to our clinic and underwent a comprehensive clinical assessment after giving informed consent. The clinical examination, as seen in Figure 1A, showed signs of severe bruising in the left periorbital region and left lower face, with associated swelling, skin discoloration, and blanching over the left nasolabial fold, upper lip, and left buccal area. Capillary refill time assessment was limited due to the bruising; however, it was prolonged (>3 seconds) in certain areas in comparison to the contralateral side, with signs suggesting ischemia, but there was no evidence of tissue necrosis. Changes in the inner buccal mucosa were also observed in Figure 1B during the intraoral examination, further suggesting ischemia.

**Figure 2:**
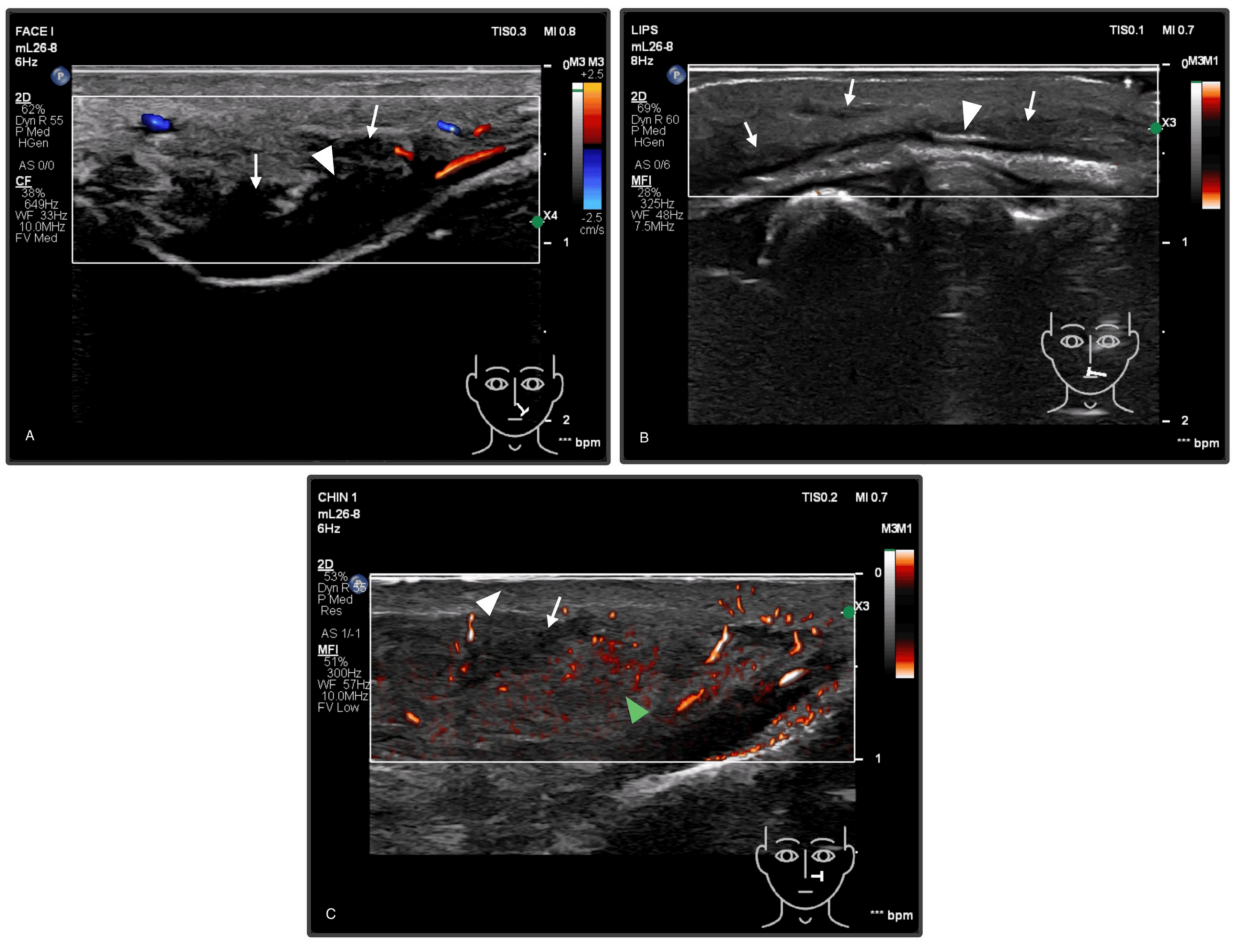
Pre-intervention grayscale, color Doppler, and microflow imaging (MFI) ultrasound findings of hyaluronic acid (HA) filler-related vascular occlusion. (A) Nasolabial region: Left angular artery showing reduced, segmental color Doppler flow (arrowhead). The adjacent anechoic heterogeneous filler material (arrow) suggests extrinsic vascular compression. (B) Upper lip: Absent vascular flow of the left superior labial artery and arterioles on MFI evaluation (arrowhead). Band-like HA filler deposits (arrow) within the subcutaneous plane of the upper lip. (C) Buccal region: Microflow imaging demonstrating a focal ill-defined hypoechoic area (arrow), consistent with residual filler, with surrounding increased vascular flow (green arrowhead) and focal reduction in perforator flow (white arrowhead) in the left buccal area.

Given the clinical concern for an underlying vascular occlusion, an ultrasound examination with Doppler was performed, using a high-resolution ultrasound system (Philips EPIQ Elite, Philips Ultrasound Inc., Bothell, Washington, USA), and with linear probes (mL26-8 and EL18-4). Multiple imaging modes were used, including B-mode, Color flow Doppler, and microflow imaging (MFI), to accurately assess vascular perfusion, demonstrating reduced Doppler flow and absent perforators in the affected areas. Even with the limited visualization due to the surrounding hematoma, ultrasound detection in Figure 2 revealed reduced perfusion and diminished blood supply in the left buccal region, left nasolabial fold, and left upper lip, as well as an absence of perforators in these areas. Further Doppler assessment confirmed vascular occlusions in the angular artery and the left superior labial artery. Anechoic deposits of HA were identified in the left piriform fossa and the upper lip, causing extrinsic compression of the left angular and the left superior labial arteries. 

Clinical and imaging findings confirmed the diagnosis of HA filler-induced vascular occlusion of the left angular and left superior labial arteries, with early ischemic changes (persistent blanching and delayed capillary refill) consistent with early stage 2 vascular occlusion [5].

**Figure 3:**
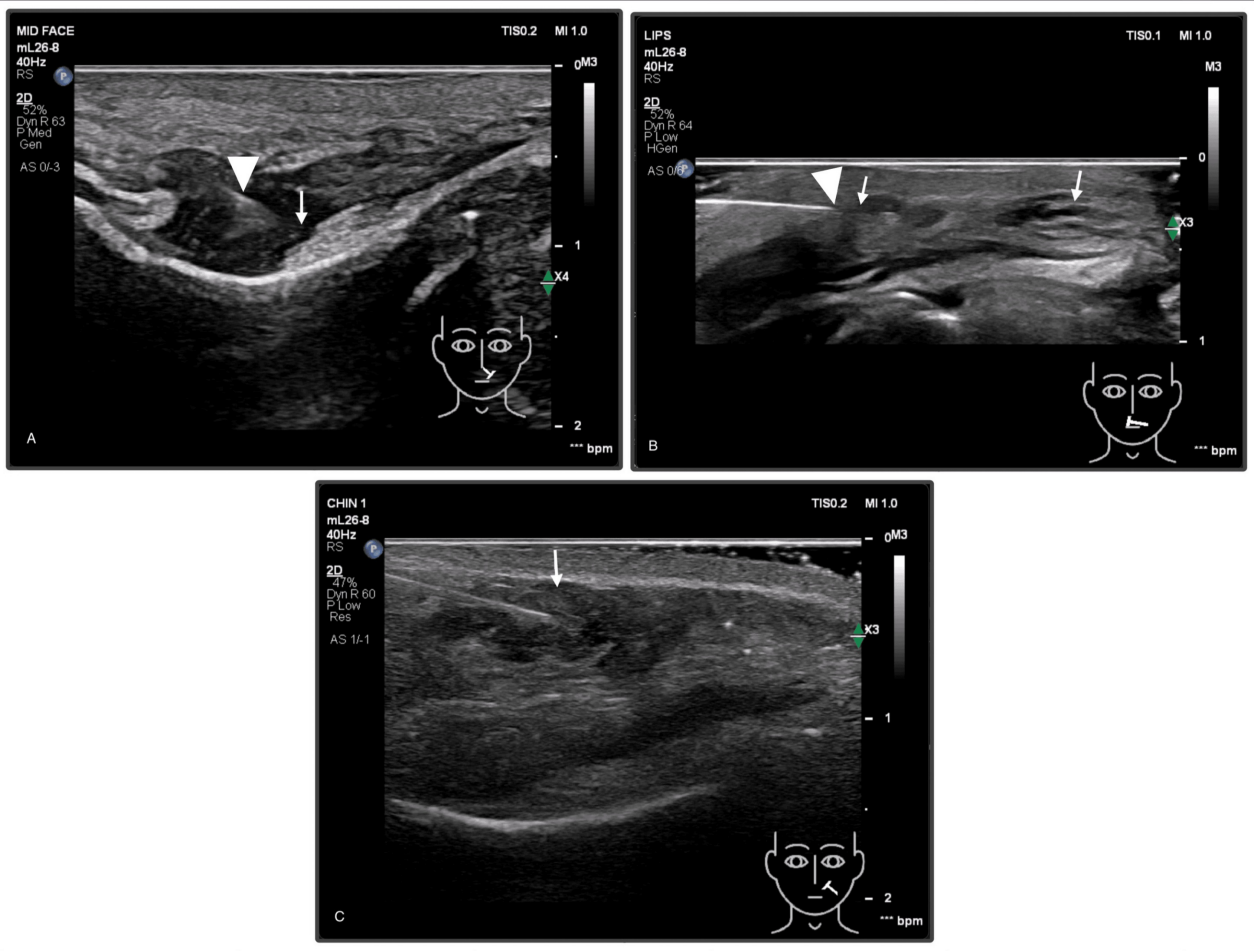
Real-time grayscale ultrasound-guided hyaluronidase injection within hyaluronic acid deposits. (A) Nasolabial region: Deep needle placement (arrowhead) demonstrating injection within the filler deposit (arrow) in the left piriform fossa. (B) Upper lip: Needle tip (arrowhead) positioned into the subcutaneous plane within the band-like filler deposit (arrow). (C) Buccal region: Superficial injection (arrow) lateral to the left angular artery targeting perforators of the left buccal area.

Immediate treatment was performed and it consisted of ultrasound-guided hyaluronidase injections to the affected areas, shown in Figure 3. Two treatment cycles with a total of 1560 IU (1 vial+, 1500 IU/vial) were administered after an allergy test for hyaluronidase was negative. The dose was distributed as 960 units to the left nasolabial fold injected directly into HA deposits within the piriform fossa, 450 units to the left upper lip into the subcutaneous plane injected within band-like HA deposits, and 150 units flooded to the left buccal area to saturate and target affected perforators [7]. Secondary measures like topical nitroglycerin spray and gentle massage were also used to promote reperfusion, which was observed immediately after.

**Figure 4:**
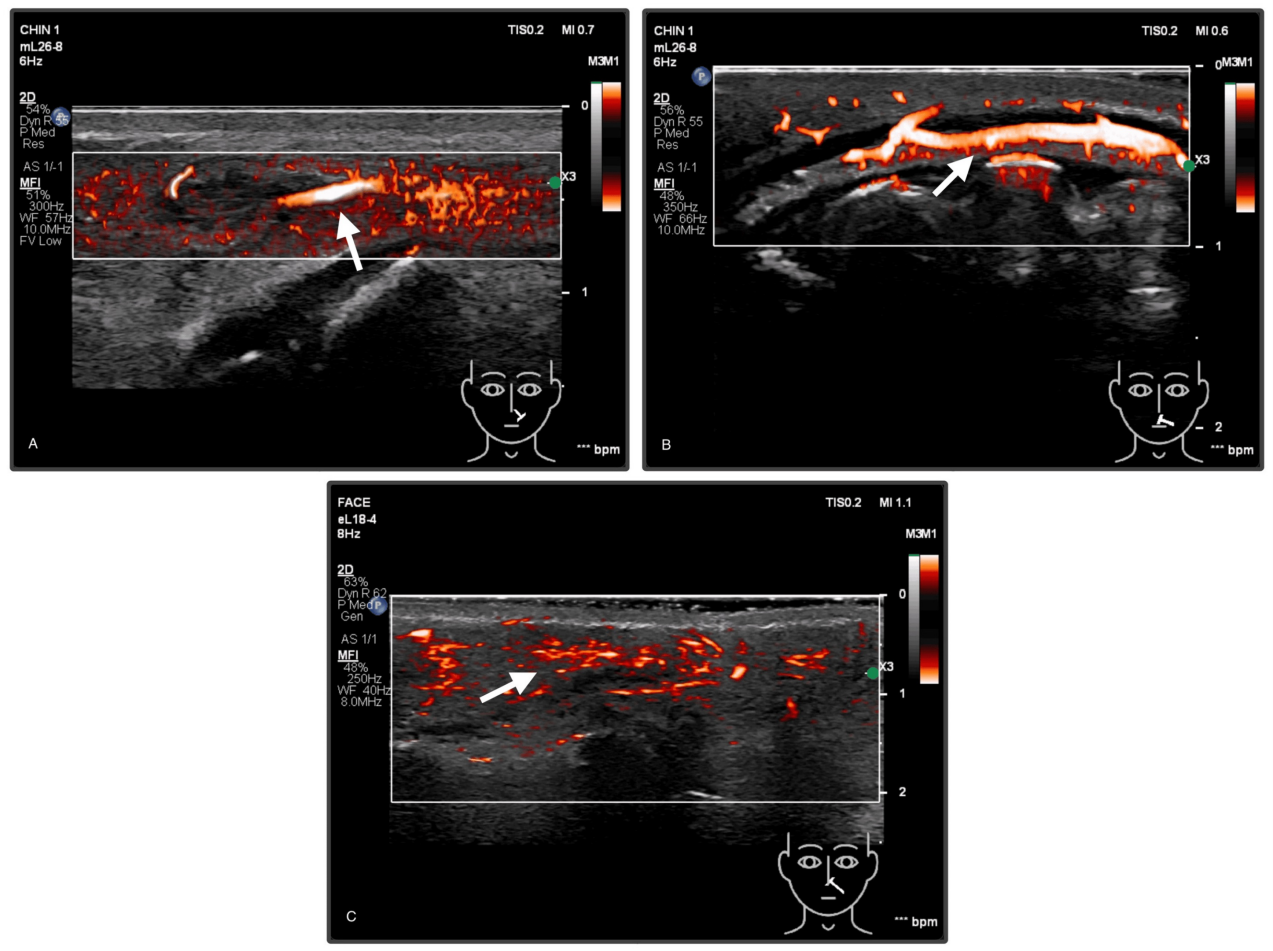
Post-intervention color Doppler and MFI ultrasound findings demonstrating restored vascular flow. (A) Nasolabial region: Restored vascular flow in the left angular artery (arrow). (B) Upper lip: Restored vascular flow within the left superior labial artery (arrow). (C) Buccal region: Improved vascular perfusion (arrow) in the left buccal area with restored perforators. MFI: Microflow imaging

Post-treatment imaging demonstrated restored vascular flow in the affected areas on color Doppler and MFI evaluation following ultrasound-guided hyaluronidase injection, shown in Figure 4. Clinical examination showed improvement in capillary refill time (three seconds) and resolution of skin blanching and ischemic signs. The patient was started on a post-vascular occlusion management plan, continuing aspirin and antibiotics, along with corticosteroids and antiviral therapy. Topical prescriptions included fusidic acid ointment, acyclovir, and topical exosomes. Furthermore, hyperbaric oxygen therapy was recommended to support oxygenation and reduce the risk of ischemic progression, and she completed three sessions, with close follow-up over the next three days to monitor her progression.

**Figure 5:**
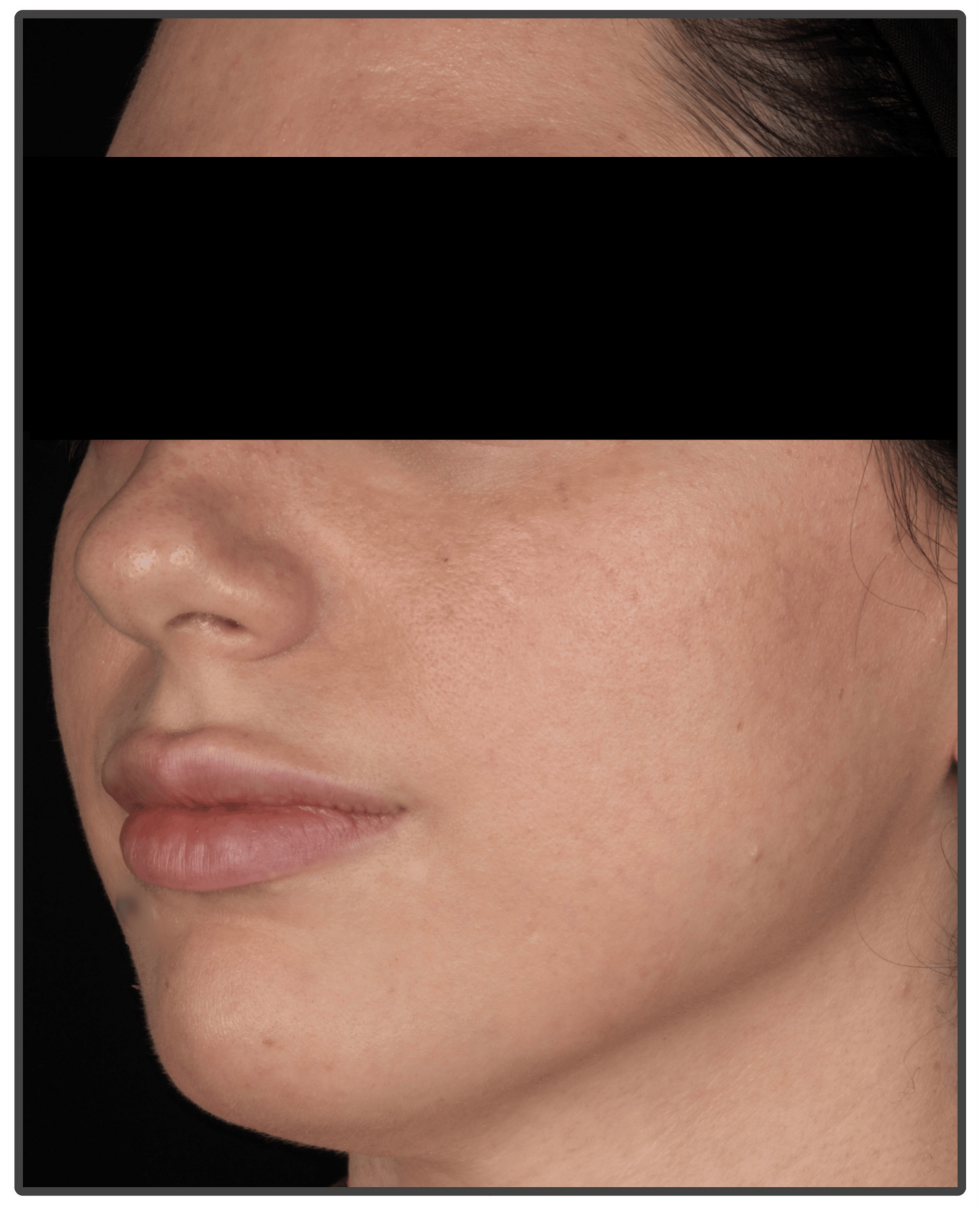
Clinical appearance at one month follow-up, demonstrating fully healed skin with no signs of permanent tissue damage.

At her one-month follow-up, the patient showed complete recovery with no residual skin changes or evidence of tissue damage, as shown in Figure 5, reflecting the positive impact of early and targeted management.

## Discussion

HA fillers are widely considered safe, but vascular occlusions remain one of the most serious complications, with risks of resulting in tissue ischemia or necrosis if they are not recognized and treated early [5,8,9]. Even though such complications are rare, their consequences can be severe, especially in anatomically high-risk areas such as the nasolabial folds [5,10]. This case highlights how vascular compromise does not always present in a clear or typical way, and how delays in diagnosis can happen when classic clinical signs are absent, thus complicating management and increasing the risk of serious outcomes.

Vascular occlusions after dermal fillers are well described in the literature, and they commonly involve branches of the facial artery such as the angular artery [9]. Early clinical signs include blanching, pain, and delayed capillary refill, and when they are left untreated, they could progress to livedo reticularis and tissue necrosis [5]. However, this case points out how these classic features can be obscured. In particular, blanching was overlooked, which delayed the suspicion of a vascular event. Pain, which is usually an important warning sign, became less reliable after lidocaine was given to the patient to reduce discomfort from the occlusions and the repeated hyaluronidase injections [9]. In addition, the development of a large hematoma introduced more challenges clinically because it obscured ischemic changes such as livedo, discoloration, and even capillary refill time, creating a masking effect. 

The pathophysiology behind vascular occlusions either involves intravascular injection, external compression by filler material, or arterial spasms, which can result in impaired blood flow, reduced perfusion, ischemia, and tissue necrosis [1,7]. In this case, more than one vessel was involved, the left angular and the left superior labial arteries, suggesting a more extensive vascular event. The involvement of both vessels may be explained by the anatomy of the facial arterial network and its anastomosis. The facial artery gives rise to the superior labial artery and continues as the angular artery along the nasolabial fold, placing injections in the piriform fossa and upper lip in close proximity to these vessels. In addition, collateral connections between the facial, infraorbital, and dorsal nasal arteries may allow spread of filler material under pressure, potentially contributing to the multi-vessel involvement and ischemic pattern observed in this case [5,6]. It is also important to note that a cannula was used during the injection, which is usually considered safe by many practitioners; however, its use does not eliminate the risk entirely, and careful techniques and safety measures remain essential [5].

A key feature of this case is the initial management approach at the referring clinic. Current guidelines emphasize the use of targeted and timely doses of hyaluronidase as the main treatment for vascular occlusions, which are usually combined with vasodilators to enhance reperfusion [3,8]. The patient received multiple high-dose cycles in a relatively short timeframe; this exaggerated protocol may have caused an increase in tissue trauma and led to hematoma formation [3]. Moreover, vascular occlusions could be assessed through affected perforators corresponding to region-specific reticulated patterns [11]. However, the pattern could not be clearly assessed in this case due to the underlying hematoma. As a result, this complicated the clinical picture and challenged the assessment by masking important signs such as livedo and progressive discoloration [9]. This case therefore illustrates that an excessive escalation of treatment may be counterproductive when it’s not supported by a clear diagnosis [3,12].

Ultrasound played a significant role in this case, both by confirming the diagnosis and guiding management, and although the clinical picture was unclear, it helped locate the hypoechoic HA deposits adjacent to the affected vessels, demonstrated reduced and absent vascular flow on Doppler evaluation, and confirmed the involvement of multiple vessels, allowing for a precise and targeted administration of hyaluronidase. In addition, reperfusion post treatment was demonstrated on both color Doppler and MFI, providing real-time confirmation of vascular restoration, which cannot be reliably assessed through clinical examination alone [6,13]. The presence of a hematoma also made imaging more challenging, as it can complicate the visualization of structures, but ultrasound remained a valuable tool, providing accuracy even in such challenging scenarios [12]. This improved treatment precision also reduced the need for unnecessary escalation. Although not performed in this case, pre-procedural vascular mapping with ultrasound may reduce risk by identifying individual vascular structures before injection, especially in high-risk areas [13,14].

From a clinical perspective, this case serves several important lessons. Early warning signs such as blanching should never be ignored and must be acted upon immediately, even in the absence of other symptoms. Pain may not always be a reliable sign, especially if lidocaine is involved in procedural or post-procedural administrations, altering and masking diagnostic features [9,15]. Excessive bruising should also be assessed carefully, as it can hide more serious underlying problems. Moreover, the use of cannulas should not create a false sense of safety, as the risk of vascular complications remains possible [5]. Finally, incorporating ultrasound into aesthetic practice offers an advantage by improving diagnostic accuracy and guiding targeted intervention, especially in complex cases [11,13,14,16,17].

This report has limitations. As a single case, findings may not be generalizable, and there is a lack of comparative data, limiting the ability to evaluate the effectiveness of this approach across multiple cases and management strategies. However, it supports the growing evidence of the use of ultrasound in improving treatment outcomes and reducing the risk of complications. 

## Conclusions

This case demonstrates how vascular occlusions can be difficult to recognize and manage when clinically obscuring factors, such as hematoma and pain-masking lidocaine, interfere with the presentation. It also shows that overly aggressive and exaggerated treatments may complicate the clinical picture rather than improve it. It highlights the risks of missed recognition of early signs of vascular occlusion and inefficient management, and how ultrasound allows for a more precise and focused treatment compared to other management protocols. This case reinforces the importance of post-injection monitoring and acting quickly and effectively when there is any suspicion of vascular compromise. Overall, adding ultrasound to routine aesthetic practice can make procedures safer and more accurate in complex cases.
